# Antimicrobial peptide GL13K-Modified titanium in the epigenetic regulation of osteoclast differentiation via H3K27me3

**DOI:** 10.3389/fbioe.2024.1497265

**Published:** 2024-10-24

**Authors:** Yuerong Gao, Yingzhen Lai, Hong Wang, Jingjing Su, Yan Chen, ShunJie Mao, Xin Guan, Yihuang Cai, Jiang Chen

**Affiliations:** ^1^ School and Hospital of Stomatology, Fujian Medical University, Fuzhou, Fujian, China; ^2^ Department of Stomatology, Engineering Research Center of Fujian University for Stomatological Biomaterials, Xiamen Medical College, Xiamen, Fujian, China; ^3^ Stomatological Hospital of Xiamen Medical College, Xiamen, Fujian, China

**Keywords:** dental implant, GL13K, osteoclastogenic differentiation, epigenetic regulation, histone methylation

## Abstract

Implant surface designs have advanced to address challenges in oral rehabilitation for healthy and compromised bone. Several studies have analyzed the effects of altering material surfaces on osteogenic differentiation. However, the crucial role of osteoclasts in osseointegration has often been overlooked. Overactive osteoclasts can compromise implant stability. In this study, we employed a silanization method to alter pure titanium to produce a surface loaded with the antimicrobial peptide GL13K that enhanced biocompatibility. Pure titanium (Ti), silanization-modified titanium, and GL13K-modified titanium (GL13K-Ti) were co-cultured with macrophages. Our findings indicated that GL13K-Ti partially inhibited osteoclastogenesis and expression of osteoclast-related genes and proteins by limiting the formation of the actin ring, an important structure for osteoclast bone resorption. Our subsequent experiments confirmed the epigenetic role in regulating this process. GL13K-Ti was found to impact the degree of methylation modifications of H3K27 in the *NFATc1* promoter region following RANKL-induced osteoclastic differentiation. In conclusion, our study unveils the potential mechanism of methylation modifications, a type of epigenetic regulatory modality, on osteoclastogenesis and activity on the surface of a material. This presents novel concepts and ideas for further broadening the clinical indications of oral implants and targeting the design of implant surfaces.

## Graphical Abstract

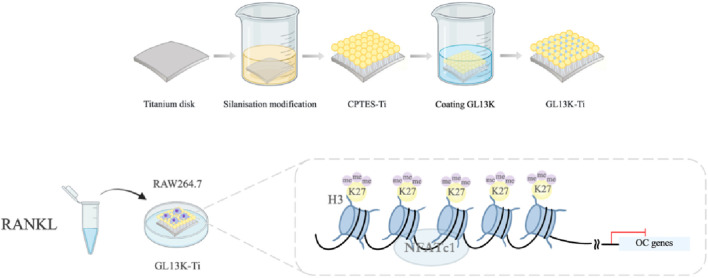

## 1 Introduction

The durable performance of implants is reliant on exceptional osseointegration and the antimicrobial characteristics of the implant material (Silveira et al., 2023; Lu et al., 2021). It is commonly accepted that augmenting the roughness and wettability of the implant substrate surface improves cell adhesion and growth, subsequently promoting better osseointegration (Jemat et al., 2015). Nevertheless, increased roughness and wettability have a direct association with bacterial retention (Kligman et al., 2021; Robles et al., 2023). GL13K is a bacterial-agglutinating peptide that originates from the human parotid secretory protein (Gorr et al., 2008). A study has reported that coatings modified with GL13K are effective at inhibiting the primary pathogens responsible for peri-implantitis and are also biocompatible with macrophages (Li et al., 2017). In our previous study, we applied a silanization method for immobilizing the antimicrobial peptide GL13K onto a titanium surface. The results demonstrated that GL13K-Ti exhibited potent bactericidal activity against *Porphyromonas gingivalis* and effectively regulated inflammatory factor secretion in the local microenvironment (Zhou et al., 2015). Additionally, it triggered the conversion of M1 macrophages to the M2 phenotype (Chen et al., 2020). Moreover, GL13K-Ti induced osteogenic and angiogenic differentiation to some extent (Zhou et al., 2021). Macrophages, which exhibit high plasticity in the microenvironment following implant placement, play a crucial role in bone regeneration (Zhang et al., 2022; Schlundt et al., 2021). Osteoclasts, as organ-specific tissue-resident macrophages, participate not only in bone resorption but also secrete related factors to collaborate with osteoblasts in the mediation of bone regeneration (Kim et al., 2020; Weivoda and Bradley, 2023). While osteoclasts can complete bone resorption in only a few weeks, osteoblasts often need months to generate a new bone (Lee et al., 2021). Hence, curbing excessive osteoclast activation on the implant surface can enhance the initial stability and preserve marginal bone levels following implantation, thereby further fostering long-term stability and a high success rate, which is particularly significant for patients with factors leading to poor healing, such as peri-implantitis, osteoporosis, and diabetes (De Oliveira et al., 2020).

An increasing number of studies support the significant role of epigenetic regulation, a mechanism that governs gene expression and cell growth and differentiation without altering the DNA sequence, in the process of osteointegration differentiation on material surfaces (Bighetti-Trevisan et al., 2022; Oton-Gonzalez et al., 2022; Cho et al., 2021; Liu et al., 2021; Tammen et al., 2013). Nevertheless, the impact of epigenetic regulation on osteoclast differentiation caused by surface modifications has seldom been studied. NFATc1 plays a crucial role as a transcription factor in the terminal differentiation process of osteoclasts (Bae et al., 2022). Histone H3 lysine trimethylation at position 27 (H3K27me3) is typically linked with gene silencing (Ma et al., 2020; Fontcuberta-PiSunyer et al., 2018). Results from chromatin immunoprecipitation (ChIP) sequencing revealed that Jumonji domain protein 3 (Jmjd3), an H3K27 demethylase, is upregulated and recruited near the *NFATc1* transcription start site during osteoclastogenesis (Yasui et al., 2011). Consequently, the degree of H3K27me3 is diminished, which activates the RANKL signaling pathway. This is consistent with previous research showing that knockdown of *Jmjd3* decreased osteoclastogenesis (Marks and Walker, 1981). Our previous studies have verified that GL13K-Ti impeded the translocation of NFATc1 into the nucleus (Zhou et al., 2021).

Based on our previous research, a GL13K-Ti surface was fabricated and characterized, with Ti and CPTES-Ti surfaces serving as controls. Following RANKL induction, we investigated the macrophage response to the GL13K-Ti surface. Additionally, we examined the level of H3K27me3 in the promoter region of the key osteoclastogenic transcription factor *NFATc1*.

In summary, this study seeks to offer fresh insights and ideas for the surface modification of implant materials and their design to aid patients with bone healing defects. It does so by investigating the influence of a GL13K-modified titanium surface on osteoclast differentiation and the potential underlying epigenetic regulation mechanisms.

## 2 Experimental procedures

### 2.1 Preparation of titanium specimens

Titanium specimens were divided into three groups: the pure titanium group (Ti), silanation group (CPTES-Ti), and antimicrobial peptide GL13K modification group (GL13K-Ti). All pure titanium specimens (99.5% purity, Alfa Aesar, USA) were of two sizes, 10 mm × 10 mm × 0.25 mm and 20 mm × 20 mm × 0.25 mm (for RT-PCR, Western blotting and ChIP assays), were polished with silicon carbide sandpapers of 600, 800, 1200, and 2000 grit in series, and then washed with acetone, absolute alcohol, and deionized water (dH_2_O) in an ultrasonic cleaner for 15 min. Subsequently, the specimens were dried naturally. The silanation and GL13K modification groups were further processed as previously described. Briefly, the titanium specimens were soaked in 5 M NaOH overnight at 60°C to form reactive -OH groups on the Ti surfaces and then placed in 7 mL of anhydrous pentane (Sigma-Aldrich), 1.2 mL of (3-chloropropyl) triethoxysilane (CPTES, Sigma-Aldrich), and 0.6 mL of diisopropylethylamine (DIEA, Sigma-Aldrich). Covalent immobilization of the GL13K peptides was accomplished by immersing silanized Ti specimens into a mixed solution with 0.1 mM GL13K (GKIIKLKASLKLL-CONH2) in 0.5 mg/mL Na_2_CO_3_ overnight. All samples were sterilized using ethylene oxide gas prior to inoculation of the RAW264.7 cells.

### 2.2 Surface characterization

The surface morphology of the Ti, CPTES-Ti, and GL13K-Ti specimens was characterized using field emission scanning electron microscopy (SUPRA 55, ZEISS, Germany, SEIR:0.8 nm) after being sprayed with gold. Distilled water (2 μL) was applied to the surface of each specimen at room temperature. The static contact angle on each image was obtained (DSA30, KRUSS, Germany). Elemental composition analysis was carried out by X-ray photoelectron spectroscopy (PHI Quantum 2000; PHI, USA). The roughness was scanned by an atomic force microscope (Cypher S, Oxford Instrument Asylum Research, England, Tip type:AC240, Tapping Mode).

### 2.3 RAW264.7 cell culture and osteoclastogenic induction

The RAW264.7 cell line was obtained from the Cell Bank of the Chinese Academy of Sciences (Shanghai, China). Cells were cultured in Dulbecco’s modified Eagle medium (DMEM, high glucose medium) supplemented with antibiotics (100 U/mL penicillin A and 100 U/mL streptomycin) and 10% fetal bovine serum (FBS), in an atmosphere of 5% CO_2_ and 95% humidity at 37°C. RAW264.7 cells were seeded at a density of 2×10^4^ cells/well (24-well dish) and 1×10^5^ cells/well (6-well dish) when they reached ∼80% confluence. After 24 h, the cell medium was replaced with osteoclastogenesis medium containing 50 ng/mL RANKL (R&D, USA). The RAW264.7 cells were differentiated for 5 days, and the medium was changed every other day.

### 2.4 Cell morphology and proliferation assay

On days 1, 3, and 5 after plating, cells attached to the samples were incubated with a Cell Counting Kit-8 (CCK-8, Dojindo, Japan) reagent. RAW264.7 cell proliferation was determined by measuring the OD value at 450 nm. The method for cell seeding and inducing osteoclast differentiation was the same as described in the previous sections. The cells were washed twice with PBS, fixed with 2.5% glutaraldehyde at 4°C overnight, and dehydrated sequentially with an ethanol gradient (50, 70, 90, and 100 vol%). After vacuum drying using hexamethyldisilazane (HMDS) and spraying with gold, the morphology of the attached RAW264.7 cells and osteoclasts was observed by scanning electron microscope (SEM) (SUPRA 55, ZEISS, Germany).

### 2.5 Tartrate-resistant acid phosphatase (TRAP) activity

Cells (1×10^5^ cells/well) were seeded onto the samples for 5 days. TRAP enzyme activity was measured with a TRAP enzyme assay kit (Beyotime, China) according to the manufacturer’s instructions. The absorbance at 405 nm was measured.

### 2.6 Immunofluorescence

The H3K27me3 protein was analyzed on day 5 by immunofluorescence with the primary rabbit monoclonal antibody anti‐H3K27me3 (ab192985, 1:1000; Abcam), followed by Alexa Fluor 488‐conjugated secondary anti‐rabbit IgG horseradish peroxidase (HRP) antibody (ab270162, 1:100; Abcam). The nuclei were stained with blue fluorescent 4′,6‐diamidino‐2‐phenylindole dihydrochloride (DAPI; D9542, 1:500; Sigma, USA). Three samples were observed, and random images were acquired as described above. The actin cytoskeleton was stained with rhodamine phalloidin (PHDR1, Cytoskeleton, USA) and the nuclei with DAPI, as described above. A confocal laser scanning microscope (CLSM; TCS SP8, Leica, Germany) was used to analyze all specimens.

### 2.7 Quantitative real-time polymerase chain reaction (qRT-PCR)

Total RNA from cells after a 5-day culture on different samples was isolated with TRIzol reagent (Sigma, USA). An ultraviolet (UV) spectrophotometer was used to measure the RNA concentration and purity, and only samples with an absorbance ratio (260/280 ratio) > 1.8 were selected for subsequent experiments. Reverse transcription of mRNA was performed by using a PrimeScript RT Master Mix 10-mL system kit (TaKaRa, Japan). qRT-PCR was carried out with a SYBR Premix Ex Taq II kit (TaKaRa, Japan) with the designed primers (Table 1) on a LightCycler 480 (Roche, Switzerland). Osteoclast marker genes, including *TRAP*, *NFATc1*, *β3 integrin*, *MMP9*, *Ctsk*, *DC-STAMP*, *Atp6v0d2*, *c-src*, and *c-Fos*, were assessed with *GAPDH* as the housekeeping gene. The level of gene expression was calculated via the 2^−ΔΔCT^ method. Three independent samples were used for each gene of interest.

**TABLE 1:** Primer Sequences Used for Quantitative real-time polymerase chain reaction.

Gene	5′–3′	Primer sequence
*TRAP*	Forward Reverse	CACTCCCACCCTGAGATTTGT CATCGTCTGCACGGTTCTG
*NFATc1*	Forward Reverse	CCGTCACATTCTGGTCCATAC TTCATTCTCCAAGTAACCGTGTAG
*β3 integrin*	Forward Reverse	AGTGCGATGACTTCTCCTGC CAGGTGTCAGTGCGTGTAGT
*MMP9*	Forward Reverse	ATGTCACTTTCCCTTCACCTTC TGCCGTCCTTATCGTAGTCA
*Ctsk*	Forward Reverse	AATTATGGCTGTGGAGGCGG TGCATTTAGCTGCCTTTGCC
*DC-STAMP*	Forward Reverse	TTGCCACTCCGCTGAATCTA GCTCTGTCGTGACCACCATA
*c-src*	Forward Reverse	TGTACGGCAGGTTCACCATC AACCTCACGGTTCACCATCC
*c-Fos*	Forward Reverse	GAGCCAGTCAAGAGCATCAG GCATAGAAGGAACCGGACAG
*Atp6v0d2*	Forward Reverse	AGTCTTACCTTGAGGCATTCTACA TCTCCCTGTCTTCTTTGCTTAGT
*GAPDH*	Forward Reverse	TGGAAAGCTGTGGCGTGATG TACTTGGCAGGTTTCTCCAGG

### 2.8 Western blotting

RAW264.7 cells induced by RANKL (50 ng/mL) were incubated in a 6-well plate for 5 days with the different materials, and then, the cells were lysed in RIPA buffer with a protease inhibitor cocktail. The cell lysates were incubated on ice for 5 min and then centrifuged (4°C, 5 min, 10,000×g). The supernatants were collected from each sample. A bicinchoninic acid (BCA) protein assay kit (Beyotime, China) was used to quantify the total protein content of the lysates. Proteins were separated by sodium dodecyl sulfate-polyacrylamide gel electrophoresis (SDS-PAGE) and transferred to polyvinylidene fluoride (PVDF) membranes (Millipore, USA). After blocking with skimmed milk (5%) in TBST (tris-buffered saline with 0.1% Tween 20) for 1 h, the membranes were incubated with rabbit primary antibodies, including TRAP, MMP9, Ctsk, NFATc1, H3K27me3, and Jmjd3 (Abcam, UK), overnight at 4°C. The blots were then visualized by chemiluminescence (Bio-Rad) after incubation with HRP-conjugated secondary antibodies. Quantity One software was employed to measure the gray value of each target protein band.

### 2.9 Chromatin immunoprecipitation (ChIP) assay

The ChIP assays were performed using a Simple ChIP^®^ Enzymatic Chromatin IP kit (#9003, CST) following the manufacturer’s protocol. Briefly, the surface of the material (2 cm × 2 cm) was inoculated with RAW264.7 cells, and after induction with RANKL (50 ng/mL) for 5 days, the protein and DNA were cross-linked with 1% formaldehyde and the nuclei were precipitated and lysed. The DNA was cut into fragments of 150–900 bp in length with micrococcal nuclease and an ultrasonic sonicator, and the shearing efficiency was verified by agarose gel electrophoresis. After adding the corresponding ChIP target protein antibody to each sample, the samples were incubated on a rotor at 4°C overnight. The chromatin was adsorbed and precipitated by protein G magnetic beads and eluted with ChIP buffer to decrosslink the chromatin, and the target DNA fragments were purified and quantitatively analyzed by real-time PCR. Primer pairs used for the *NFATc1* promoter were (forward) 5′-GAA​GTG​GTA​GCC​CAC​GTG​AT-3′ and (reverse) 5′-TCT​TGG​CAC​CAC​ATA​AAC​CA-3’.

### 2.10 Statistical analysis

All the data were analyzed by using GraphPad Prism software V.9.3.1. The means and standard deviations were recorded and analyzed by one-way analysis of variance (ANOVA), and the ChIP assay results were analyzed by a *t*-test (*p* < 0.05). Where applicable, a Tukey’s honestly significant difference test was used as a *post hoc* test. *p* < 0.05 was considered statistically significant.

## 3 Results

### 3.1 Characterization of titanium specimens with different surfaces

**FIGURE 1:**
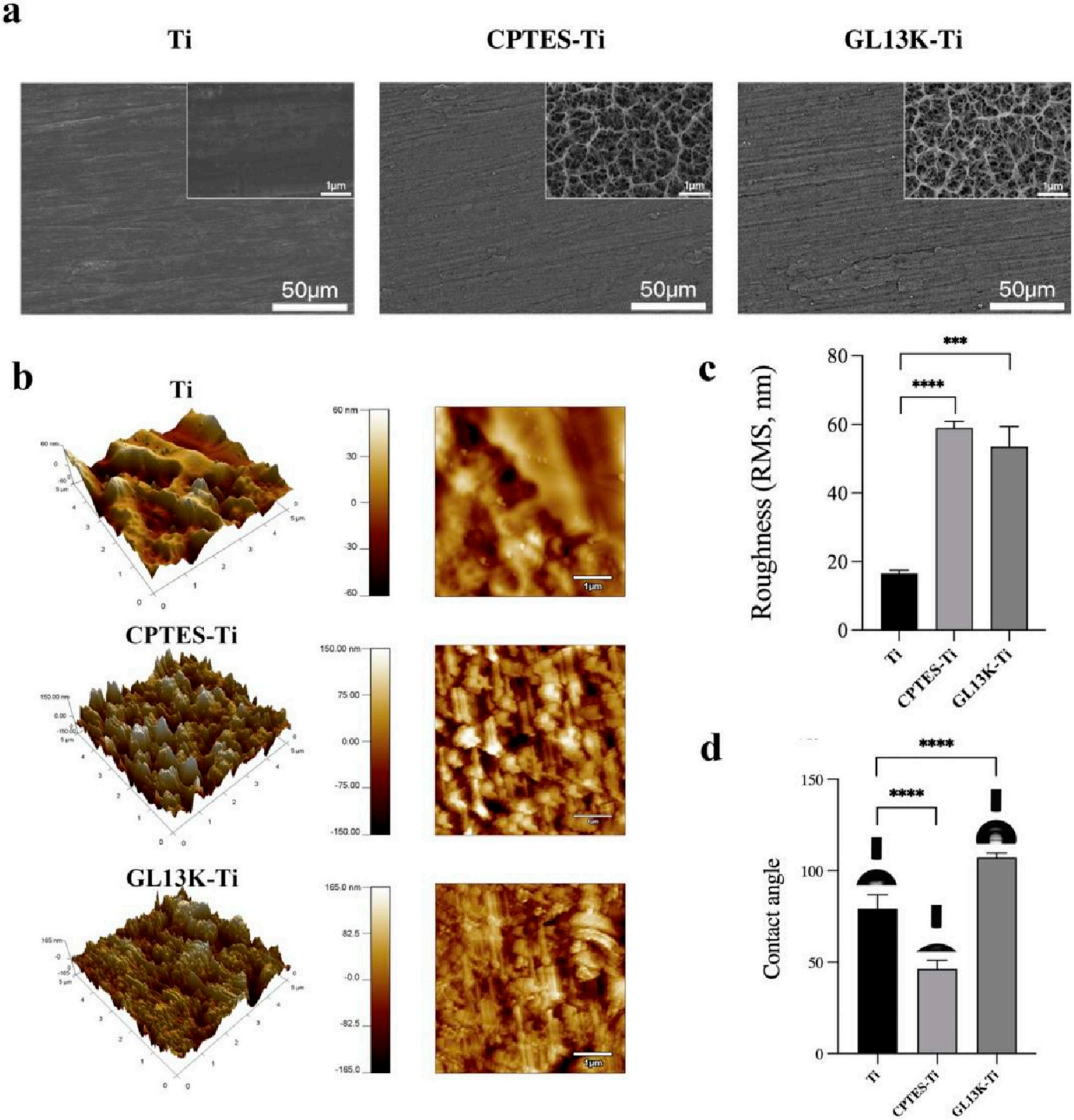
Physical surface characteristics of the materials. **(A)** Surface micromorphology of the samples, as observed via scanning electron microscope (500 and 2 × 10^4^ magnification). **(B)** Surface nanomorphology and nanoroughness, as observed via atomic force microscopy. **(C)** Root-mean-square (RMS) roughness. **(D)** Hydrophilicity of the samples. (Ti refers to pure titanium; CPTES-Ti refers to silanization-modified titanium; and GL13K-Ti refers to titanium further coated with the antimicrobial peptide GL13K after the silanization modification.)

The surface morphology of each material group was viewed using a field emission scanning electron microscope, and the findings are presented in Figure 1a. The Ti surface appeared smooth and flat with a polished texture. In contrast, the CPTES-Ti surface manifested a porous, loose structure. Similarly, the surface of GL13K-Ti exhibited irregular porosity, indicative of alkali etching. As shown in Figure 1b and 1c, the surface morphology and the average roughness of the three material groups were observed using atomic force microscopy. The results indicated a significant increase in roughness on the material surfaces modified with silanization and the antimicrobial peptide GL13K compared to Ti (*p* < 0.001). However, no significant difference was observed between the CPTES-Ti and GL13K-Ti groups (*p* = 0.35).

The hydrophilicity of the surfaces of the three material groups was analyzed using a contact angle meter (Figure 1D). On the Ti surface, the contact angle was 79.2° ± 6.8°; on the CPTES-Ti surface, the contact angle decreased to 46.3° ± 4.3°; and on the GL13K-Ti surface, the contact angle increased to 107.2° ± 2.2°, indicating hydrophobicity. The differences among the groups were statistically significant (*p* < 0.05) and therefore important.

### 3.2 Effect of GL13K-modified titanium on osteoclastogenesis

#### 3.2.1 RAW264.7 cell adhesion and proliferation on GL13K-modified titanium

**FIGURE 2:**
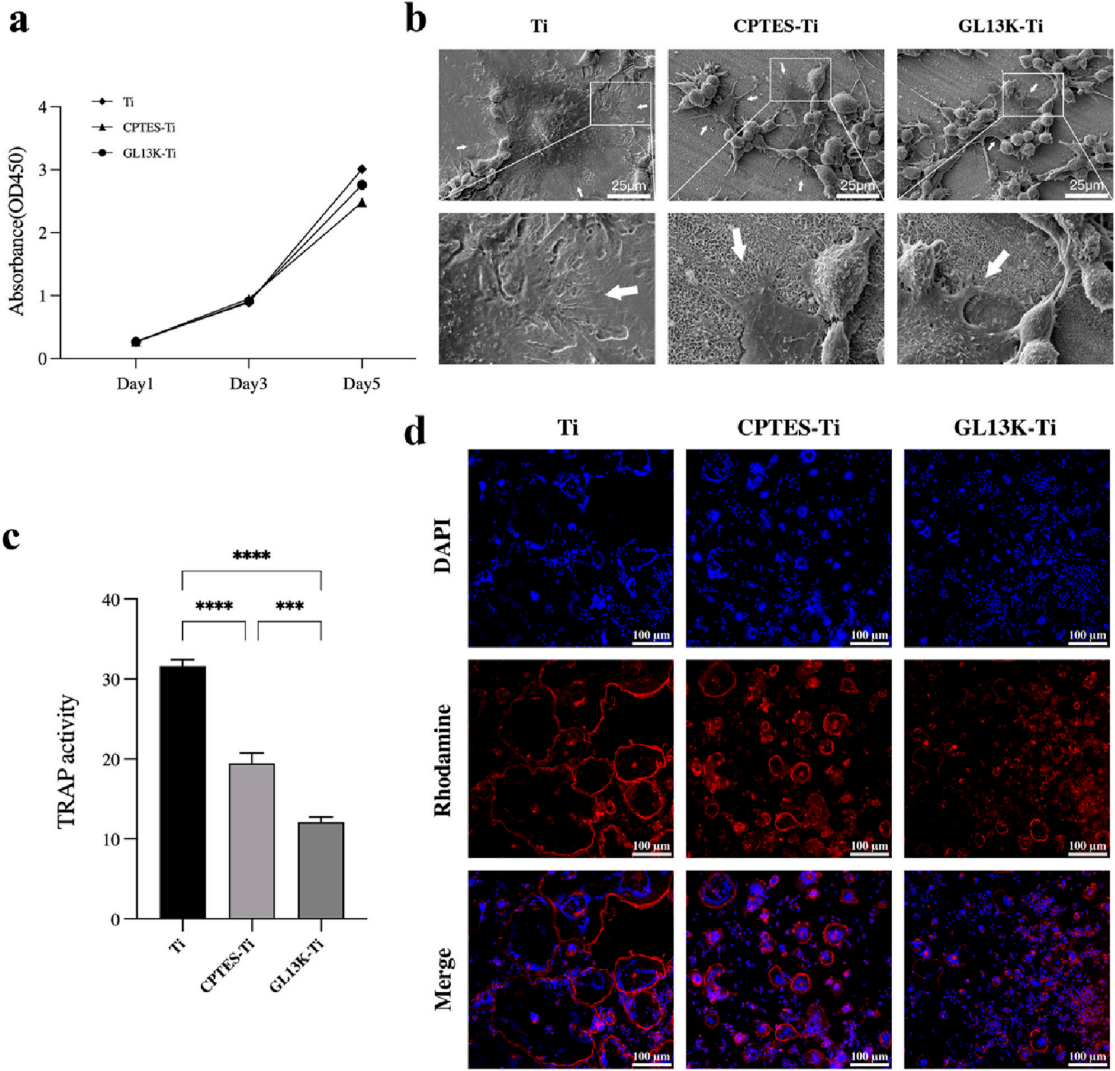
RANKL-stimulated RAW264.7 cell osteoclastogenic differentiation on different sample surfaces. **(A)** CCK-8 results of RAW264.7 cells cultured on the surface of Ti, CPTES-Ti and GL13K-Ti. **(B)** SEM images of osteoclasts on the surface of the different materials. **(C)** Results of the tartrate-resistant acid phosphatase activity assay (***: *p* < 0.0005; ****: *p* < 0.0001). **(D)** Effects of the three materials on the actin ring of osteoclasts.

The CCK-8 assay provided a quantitative result for RAW264.7 cell proliferation (Figure 2A). For RAW264.7 cell proliferation, the growth followed a logarithmic proliferation curve on all surfaces, and there was no difference between the surfaces of GL13K-modified titanium and pure titanium after 1, 3, and 5 days. It was initially determined that titanium surfaces modified with the antimicrobial peptide GL13K were essentially noncytotoxic to macrophages.

#### 3.2.2 SEM

The morphology and fine structure of the adhered cells were observed by SEM. Bone resorption by osteoclasts depends on a highly acidified microenvironment isolated from the external environment. The use of scanning electron microscopy revealed the size, shape, and adhesive characteristics of osteoclasts on the surfaces of the three materials after 5 days of RANKL induction. The range of osteoclast spread in each group is indicated by the white arrows in Figure 2B. The osteoclasts that developed on the surface of Ti material were large in size, with a dome-shaped center surrounded by numerous delicate adhesion filaments that attached to the surface of the material, forming a ring-shaped edge closure zone. This entire structure resembled the appearance of a fried egg. The macrophages present on the surface of CPTES-Ti and GL13K-Ti exhibited a different state of fusion compared to those on the Ti surface. On the Ti surface of the control group, a significant number of macrophages, which were small in size and attached to the surface with irregular lamellar pseudopods, enclosed a few osteoclasts. The marginal closure zone that is crucial for osteoclastic resorptive activity was disrupted.

#### 3.2.3 TRAP activity assay

The TRAP activity assay showed a strong correlation with osteoclast formation. Thus, a semi-quantitative analysis of TRAP activity can partially reflect the count of osteoclasts. The TRAP activity assay was carried out on macrophage-osteoclasts cultured on the surfaces of the three groups of materials, and the results are presented in Figure 2C. The highest TRAP activity was observed in the Ti group. In comparison, the TRAP activity was reduced on the surfaces of CPTES-Ti and GL13K-Ti. There was a statistically significant difference between the two groups (*p* < 0.05).

#### 3.2.4 Actin ring assay

The formation of actin rings is often used as a crucial indicator for identifying osteoclast survival and bone resorption activity. In this study, RAW264.7 cells were seeded onto the surface of three different materials and stimulated with 50 ng/mL RANKL for 5 days. The findings were then examined using a confocal laser microscope, as illustrated in Figure 2D. The osteoclasts that were formed on the Ti surface were of considerable size, and their nuclei varied in number from a few to tens. The actin rings were whole and uninterrupted. In contrast, smaller osteoclasts with thin, discontinuous actin rings were formed on CPTES-Ti relative to Ti. Similarly, the GL13K-Ti group generated notably fewer osteoclasts than the other two groups, and with the lowest volume. The actin ring structure was discontinuous, and the distribution appeared cluster-like.

#### 3.2.5 RT-qPCR

**FIGURE 3:**
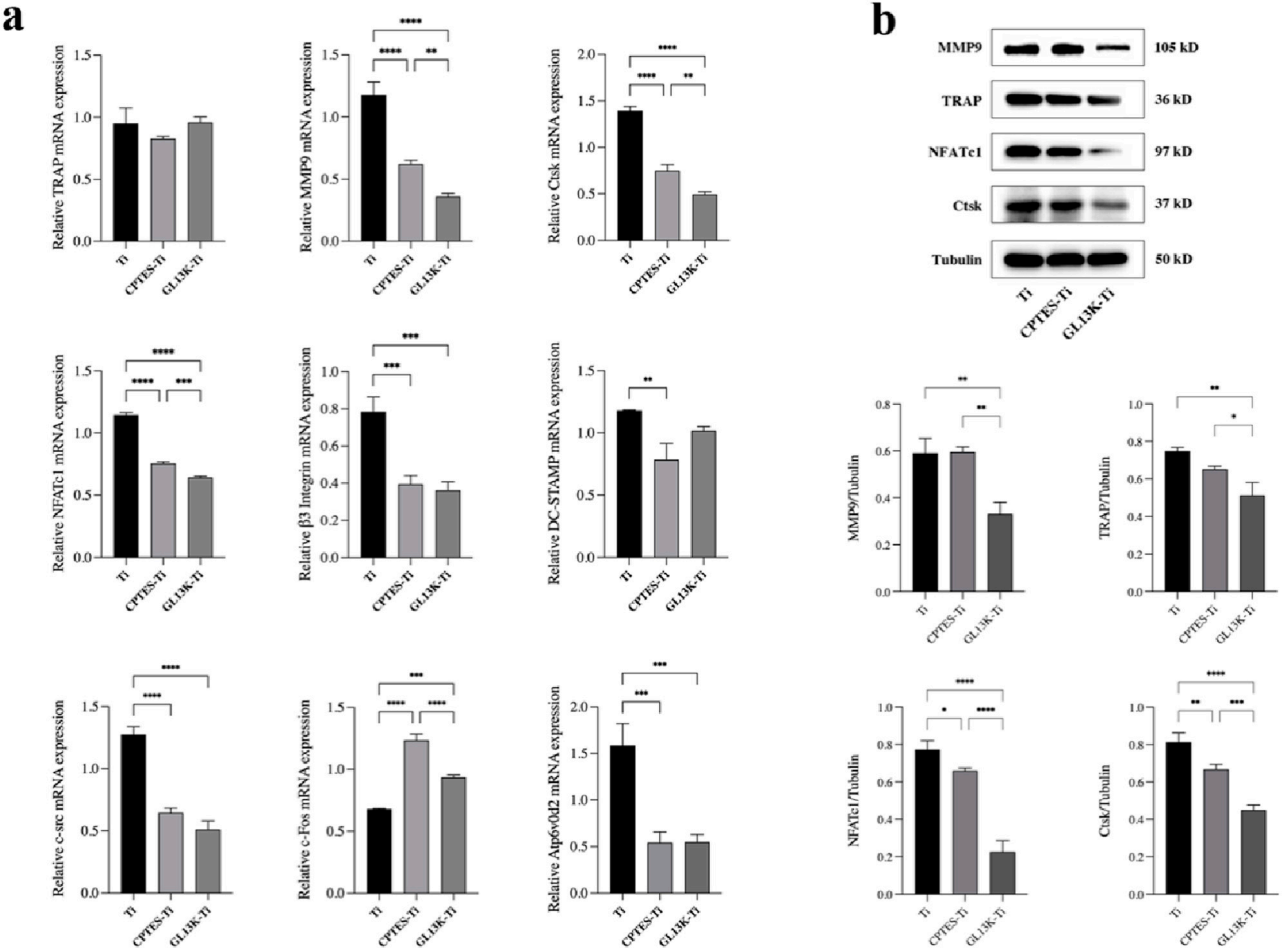
**(A)** Expression levels of osteoclast-related genes on the surface of Ti, CPTES-Ti and GL13K-Ti (**: *p* < 0.005; ***: *p* < 0.0005; ****: *p* < 0.0001). **(B)** Quantitative detection of the expression of osteoclast-related proteins on the surface of the materials (*: *p* < 0.05; **: *p* < 0.005; ***: *p* < 0.0005; ****: *p* < 0.0001). (Ti refers to pure titanium; CPTES-Ti refers to silanization-modified titanium; and GL13K-Ti refers to titanium further coated with the antimicrobial peptide GL13K after the silanization modification.)

RAW264.7 cells were co-cultured separately with each group of materials. The mRNA expression of related genes was evaluated after the addition of RANKL-induced differentiation for 5 days (Figure 3A). Compared to Ti, GL13K-Ti showed a significant reduction (*p* < 0.05) in the expression of *NFATc1*, *β3 integrin*, *MMP9*, *Ctsk*, *At6v0d2*, and *c-src*. However, there was no significant difference (*p* > 0.05) in the expression of TRAP on the surface of the three groups of materials. Regarding *c-Fos*, both the CPTES-Ti and GL13K-Ti groups showed a significantly (*p* < 0.05) higher expression than did the Ti group. Overall, GL13K-Ti significantly downregulated the mRNA expression of osteoclast-related genes, thereby inhibiting osteoclastogenesis.

#### 3.2.6 Western blot

RAW264.7 cells were inoculated onto the surface of the three groups of materials. After RANKL induction for 5 days, the total protein was extracted to quantify the expression of bone resorption-related genes at the translational level. The results are shown in Figure 3B. Consistent with the RT-qPCR findings, the expression of bone resorption-related proteins, such as MMP9, TRAP, NFATc1, and Ctsk, was downregulated in both the CPTES-Ti and GL13K-Ti groups compared to the Ti group (*p* < 0.05). Notably, GL13K-Ti induced significant downregulation of NFATc1 (*p* < 0.0001).

### 3.3 Epigenetic regulation of GL13K-modified titanium on osteoclastogenesis

To study the mechanism by which GL13K-Ti hindered osteoclastic differentiation, we assessed the H3K27me3 level using CLSM, Western blot, and chromatin immunoprecipitation techniques.

#### 3.3.1 CLSM

**FIGURE 4:**
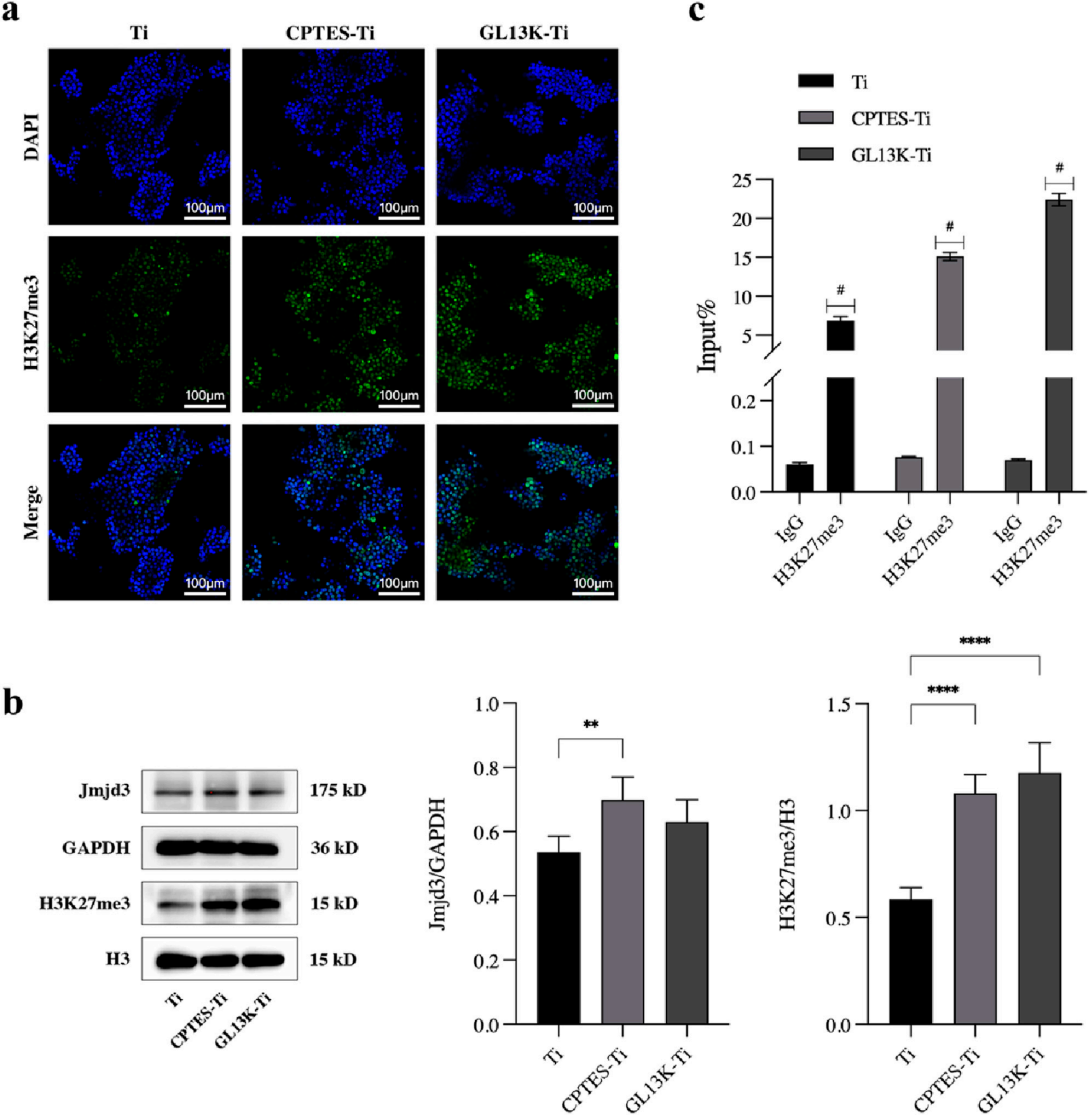
Effect of epigenetic regulation on osteoclastic differentiation by the GL13K-modified surface. **(A)** Immunofluorescence staining of H3K4 trimethylation in RAW264.7 cells cultured on different surfaces. **(B)** Protein expression of H3K27me3 and demethylase Jmjd3 on day 5 after osteoclast induction (**: *p* < 0.005; ****: *p* < 0.0001). **(C)** ChIP-qPCR assay of the trimethylation level of H3K27 on the promoter of *NFATc1* (#: *p* < 0.0001). (Ti refers to pure titanium; CPTES-Ti refers to silanization-modified titanium; and GL13K-Ti refers to titanium further coated with the antimicrobial peptide GL13K after the silanization modification.)

The nuclear localization of H3K27me3 was observed through immunofluorescence staining, and the outcome is shown in Figure 4A. When compared with Ti, the fluorescence of H3K27me3 appeared to increase in the CPTES-Ti and GL13K-Ti groups. The GL13K-Ti group depicted the most prominent green fluorescence, suggesting that the GL13K-Ti surface could have raised the modification level of H3K27me3.

#### 3.3.2 Western blot

To investigate the epigenetic regulatory mechanism of the GL13K-Ti surface inhibition of macrophage osteoclastic differentiation, the protein expression of H3K27me3 and the demethylase Jmjd3 was examined in cells on each material group using H3 and GAPDH as controls. The results are presented in Figure 4B. H3K27me3 expression was upregulated on both CPTES-Ti and GL13K-Ti surfaces compared with the control Ti, and the difference was statistically significant (*p* < 0.0001). However, Jmjd3 expression was slightly increased in the CPTES-Ti and GL13K-Ti groups, but there was no statistically significant difference between GL13K-Ti and Ti (*p* > 0.05).

#### 3.3.3 ChIP-qPCR

We conducted chromatin immunoprecipitation to determine the overall level of H3K27me3 in the *NFATc1* promoter region. The results indicated that following induction of osteoclastic differentiation in RAW264.7 cells, the level of H3K27me3 on the *NFATc1* promoter was significantly higher in the GL13K-Ti group compared to that of the Ti and CPTES-Ti groups (Figure 4C).

## 4 Discussion

### 4.1 Effects of the GL13K-modified surface on osteoclastogenesis

The elevated rate of dental implant failure has led to the development of novel materials to improve long-term retention (Souza et al., 2021; Stich et al., 2022). Most current research still focuses on enhancing osteoblast adhesion and proliferation (Wang et al., 2023; Wang et al., 2022; Ji et al., 2023; Huo and Yue, 2020). However, to maintain the balance between osteogenic and osteoclastic activities in the vicinity of the implant and increase its longevity, it is essential to assess the potential of biomaterials to inhibit osteoclast differentiation. Our previous research confirmed the efficacy of GL13K-Ti in preventing NFATc1 nuclear translocation. For this reason, the present study investigated the impact of GL13K-Ti on macrophage osteoclastic differentiation.

Osteoclast activity is affected by the physicochemical characteristics of the biomaterial, particularly the surface structure (Chen F. et al., 2019). A study (Sommer et al., 2005) conducted by Sommer et al. demonstrated that rough surfaces led to a higher number of induced osteoclasts compared to smooth surfaces. However, different material compositions had no significant effect on osteoclast formation. Another study showed that the expression of osteoclastic markers was increased in response to RANKL stimulation in a roughness-dependent manner, after different grits of sandpaper were applied to titanium discs that were then cultured with RAW264.7 cells (Makihira et al., 2007). When osteoclast precursors differentiate into mature osteoclasts, they create a dynamic cluster of F-actin-rich adhesion structures known as peduncles, which are intertwined to form closely packed superstructures referred to as actin rings (Zhang et al., 2018). This portion of the plasma membrane creates an acidic “sealing zone” known as ruffles, encircled by rings, which is ideal for the secretion of MMP9 and Ctsk by osteoclasts and for the breakdown of organic substrates (Han et al., 2019). The impact of surface roughness on the development of bone-forming cells is not yet clear because of inconsistent experimental conditions, including the source and method of cell culture, as well as the complex manner by which cells perceive the material on which they grow (He et al., 2022). In this study, we used scanning electron microscopy and atomic force microscopy to observe that CPTES-Ti and GL13K-Ti surfaces formed an uneven porous structure, resulting in an increase in roughness compared to Ti. We found using laser confocal microscopy that the actin rings on the surfaces of CPTES-Ti and GL13K-Ti were somewhat damaged. Considering that the formation of the “seal area” is vital for osteoclast performance and is altered by the nature of the surface (Chellaiah et al., 2020), it is thought that the suppression of osteoclasts on CPTES-Ti and GL13K-Ti could be linked to the unevenness of their surfaces, which hindered the formation of a working seal zone.

Antimicrobial substances are often positively charged (Mahlapuu et al., 2016). We examined the contact angle of the three materials and observed that the GL13K-Ti group had hydrophobic qualities, evidenced by the increased contact angle. This demonstrated that titanium treated with the antimicrobial substance GL13K developed a hydrophobic and positively charged surface. Previous research indicated that surfaces that are anionic and hydrophilic inhibit the adhesion and fusion of monocyte macrophages (Brodbeck et al., 2002), whereas surfaces that are cationic and hydrophobic promote macrophage adhesion and are essential for macrophages to effectively phagocytose (Da Silva Domingues et al., 2015). It is often believed that surfaces that are hydrophilic help cells adhere because they encourage proteins to arrange in an orderly manner. However, some reports disagree with this conclusion, and it is difficult to state with certainty if hydrophilic or hydrophobic surfaces are better for protein and cell adhesion.

In the presence of M-CSF, RANKL attaches to the RANK receptor on the surface of osteoclast precursor cells. This triggers downstream targets and cascade reactions that activate transcription factors, such as NFATc1, which then begin expressing osteoclast-related genes (Sapkota et al., 2018). Bone resorption relies on an extremely acidic environment that is isolated from its surroundings (Steffi et al., 2018). This environment usually contains an actin ring encircling the folded edges, creating a limited zone where matrix metalloproteinase 9 (MMP9) and histone enzyme K (Ctsk) decompose the natural matrix dominated by type I collagen fibers (Zou and Teitelbaum, 2010). Thus, NFATc1, MMP9, and Ctsk play crucial roles in facilitating the bone resorption functions of osteoclasts. Consistent with this understanding, our research findings revealed a significant decline in the RNA expression levels of these genes in the GL13K-Ti group (*p* < 0.05). Furthermore, the expression of *c-src*, *β3 integrin*, and *Atp6v0d2* genes that play a crucial role in cytoskeletal rearrangement during osteoclast fusion, actin ring and sealing zone formation, and maturation of osteoclasts (Dai et al., 2022; Uehara et al., 2019; Mo et al., 2020; Matsubara et al., 2021), was significantly downregulated by the GL13K-Ti surface (*p* < 0.05). This indicated that the GL13K-Ti surface partially hindered osteoclastic differentiation. Interestingly, the expression of individual genes at the RNA level exhibited dissimilar levels. DC-STAMP is a key regulator of osteoclast fusion and cell fusion. Research indicates that cells lacking DC-STAMP are incapable of combining into multinucleated osteoclasts (Zou et al., 2021; Takagi et al., 2017). The GL13K-Ti group showed slightly reduced expression of DC-STAMP in comparison to Ti, but there was no significant difference (*p* > 0.05). This indicated that the GL13K-modified titanium surface inhibition of the osteoclasts was not predominantly achieved through cell fusion inhibition. Furthermore, our research indicated a higher level of *c-Fos* gene expression in the GL13K-Ti group compared to the control Ti group. These findings can be explained by the involvement of the proto-oncogene *c-Fos* in the development of osteoclasts via its expression product (Chen K. et al., 2019), c-Fos protein, which is mainly regulated at the post-transcriptional level. We then examined the protein levels of TRAP, MMP9, NFATc1, and Ctsk and confirmed that GL13K-Ti decreased the protein levels of these genes. The expression of the osteoclast-associated transcription factor NFATc1 was significantly downregulated.

In brief, pure titanium that was modified with the antimicrobial peptide GL13K generated a rough, hydrophobic surface, which exhibited inhibitory effects on osteoclastogenesis. This may be attributed to the material surface reducing the expression of relevant genes and proteins. In addition, the irregular basal interface restricted the formation of osteoclast closure zones.

### 4.2 Effect of epigenetic regulation on osteoclastic differentiation of the GL13K-modified surface

Epigenetic regulation of gene expression and processes of cell growth and differentiation has been increasingly demonstrated to have a pivotal role in bone remodeling and regeneration. Cho et al. explained the epigenetic regulatory mechanisms that underlie the elevated osteogenic potential of SLA surfaces. They achieved this by co-culturing MC3T3-E1 preosteoblasts with different materials (Cho et al., 2021). Bighetti-Trevisan et al. demonstrated that a modified material surface reduced the damage caused by osteoclasts on osteoblast differentiation by measuring the degree of histone methylation modification and methyltransferase expression (Bighetti-Trevisan et al., 2022). Several other studies have also investigated the epigenetic regulatory mechanisms through which modified material surfaces promote osteogenic differentiation (Liu et al., 2021; Zheng et al., 2018). However, there is a lack of research on the influence of epigenetic regulatory mechanisms on biomaterial surfaces in osteoclastic differentiation. Our study uncovered a possible epigenetic mechanism through which GL13K-Ti suppressed the excessive activation of osteoclasts. NFATc1 is an indispensable transcription factor for osteoclast differentiation *in vivo* and *in vitro* (Kim and Kim, 2014). It is engaged in regulating the expression of osteoclast-specific genes, including *TRAP* and *Ctsk*. A study showed that embryonic stem cells lacking the *NFATc1* gene were unable to differentiate into osteoclasts when exposed to RANKL stimulation (Takayanagi et al., 2002). In addition, it was found that ectopic expression of NFATc1 led to efficient differentiation of osteoclast precursor cells, a process that partially reversed the inhibition of the RANKL pathway (Choi et al., 2014). Thus, comprehending the molecular regulatory mechanism of NFATc1 in osteoclasts could offer novel therapeutic approaches for osteoclast hyperactivation on implant surfaces. In our previous investigation, it was discovered that GL13K-Ti, in contrast to Ti, hampered the translocation of NFATc1 into the nucleus and transcription of associated downstream genes (Zhou et al., 2021). H3K27me3 is a prominent epigenetic controller of gene expression in cell differentiation and organism development. It has been demonstrated to exert a suppressive effect on osteoclastogenesis regulation (Zhu et al., 2022). The *NFATc1* gene promoter region in osteoclast precursor cells showed high levels of H3K27me3 enrichment, which was significantly reduced after RANKL stimulation (Kurotaki et al., 2020). The observed outcome may be explained by osteoclasts expressing the H3K27-specific demethylase Jmjd3 while differentiating from macrophages. Jmjd3 binds to the promoter region of the *NFATc1* gene and reduces the trimethylation level of H3K27, leading to elevated expression of *NFATc1* and its associated genes (Yasui et al., 2011). In the present study, objective findings indicated a stronger H3K27me3 fluorescence in the GL13K-Ti group compared to the control Ti group. The increased expression level of H3K27me3 protein was further validated via Western blot analysis. To investigate the association between H3K27me3 and the promoter region of *NFATc1*, ChIP-qPCR validation was performed. Our findings indicated that H3K27me3 was more abundantly enriched in the *NFATc1* promoter region on the surface of GL13K-Ti than on the surface of Ti. The dissimilarity between the groups was statistically significant (*p* < 0.05). Interestingly, the expression of the demethylase Jmjd3 was slightly elevated in the GL13K-Ti group compared to the Ti group. However, there was no statistically significant difference between the two groups (*p* > 0.05). This could be attributed to enzymes other than Jmjd3 that have an effect on the regulation of H3K27me3 modification levels. Histone methylation is only one of many modifications in the extensive epigenetic regulatory system, with dynamic regulation by both methyltransferases and demethylases. A comprehensive understanding of the epigenetic mechanism of action requires further research. In this regard, a combination of ChIP-seq and DNA microarrays would be useful in identifying new therapeutic targets.

In conclusion, it appears from our results that the GL13K-Ti surface hindered osteoclastogenesis by obstructing H3K27me3 demethylation in the promoter region of *NFATc1* during RANKL induction, leading to decreased expression of *NFATc1*.

## 5 Conclusion

In this study, we investigated how a surface modification of Ti with the antimicrobial peptide GL13K affected osteoclastic differentiation. Our results revealed that GL13K-Ti reduced mRNA and protein expression of the transcription factor NFATc1, as well as certain osteoclast-associated genes. Additionally, GK13K-Ti interfered with the actin ring integrity in osteoclasts, which led to osteoclastogenesis inhibition. Exploring the mechanism, our findings indicated that GL13K-Ti inhibited the demethylation process of H3K27me3 within the *NFATc1* promoter region. This resulted in a relative increase in the modification level of H3K27me3 and a subsequent decrease in the expression of *NFATc1*, which is a crucial transcription factor for osteoclast differentiation. To conclude, our study suggests potential for expanding the range of implant indications and enhancing osseointegration for patients experiencing inadequate bone healing post-implantation.

## Data Availability

The raw data supporting the conclusions of this article will be made available by the authors, without undue reservation.
